# Antiviral therapy in management of COVID-19: a systematic review on current evidence

**Published:** 2020-04-06

**Authors:** Mahmoud Yousefifard, Alireza Zali, Kosar Mohamed Ali, Arian Madani Neishaboori, Afshin Zarghi, Mostafa Hosseini, Saeed Safari

**Affiliations:** 1 Physiology Research Center, Iran University of Medical Sciences, Tehran, Iran.; 2 Functional Neurosurgery Research Center, Shohada Tajrish Neurosurgical Comprehensive Center of Excellence, Shahid Beheshti University of Medical Sciences, Tehran, Iran.; 3 College of medicine, University of Sulaimani, Sulaimani, Iraq.; 4 Department of Medicinal Chemistry, School of Pharmacy, Shahid Beheshti University of Medical Sciences, Tehran, Iran.; 5 Pediatric Chronic Kidney Disease Research Center, Tehran University of Medical Sciences, Tehran Iran.; 6 Department of Epidemiology and Biostatistics, School of Public Health, Tehran University of Medical Sciences, Tehran, Iran.; 7 Proteomics Research Center, Shahid Beheshti University of Medical Sciences, Tehran, Iran.; 8 Emergency Department, Shohadye Tajrish Hospital, Shahid Beheshti University of Medical Sciences, Tehran, Iran.

**Keywords:** COVID-19, Treatment, Antiviral Therapy

## Abstract

**Background:**

The purpose of the current systematic review is to evaluate the efficacy of antiviral therapies in treatment of COVID-19. In addition, clinical trials on the efficacy of antiviral therapies in the management of Severe Acute Respiratory Syndrome coronavirus (SARS-Cov) or Middle East Respiratory Syndrome coronavirus (MERS-CoV) have also been reviewed, in order to identify potential treatment options for COVID-19.

**Method:**

An extensive search was performed in Medline, Embase, Scopus, Web of Science and CENTRAL databases until the end of March 15, 2020. Two independent researchers performed the screening, and finally the related studies were included.

**Results:**

Only one clinical trial on the efficacy of antiviral therapy in management of COVID-19 was found. The results depicted that adding Lopinavir-Ritonavir to the standard treatment regimen of patients with severe COVID-19 has no benefits. Moreover, 21 case-series and case-report studies reported the prescription of antiviral agents in COVID-19, none of which can be used to determine the efficacy of antiviral therapies in confronting COVID-19. In addition, no clinical trials were found to be performed on the efficacy of antiviral agents in the management of SARS-CoV and MERS-CoV.

**Conclusion:**

The current evidence impede researchers from proposing an appropriate antiviral therapy against COVID-19, making the current situation a serious concern for international organizations such as World Health Organization (WHO). In the time of the current pandemic and future epidemics, organizations such as WHO should pursue more proactive actions and plan well-designed clinical trials so that their results can be used in managing future epidemics.

## Introduction

In the late 2019, a novel type of coronavirus emerged from Wuhan, China, causing patients to show pneumonia-like symptoms (1, 2). Later on, the virus spread around the world, and the World Health Organization announced a COVID-19 pandemic in March 2020. By March 17, 2020, 190,000 COVID-19 cases and approximately 7500 deaths from the virus were identified (3). This large number of infected patients in only three months since the first reported case of COVID-19 demonstrates that the disease is extremely contagious.

COVID-19 is caused by Severe Acute Respiratory Syndrome coronavirus 2 (SARS-CoV-2), which belongs to the family Coronaviridae. The family that has been responsible for two other viral outbreaks in recent years, the first of which was in 2003 caused by Severe Acute Respiratory Syndrome coronavirus (SARS-CoV) (4). The second outbreak occurred in 2012 and 2015 due to the spread of Middle East respiratory syndrome coronavirus (MERS-CoV), another virus from the same family (5, 6). These two epidemics infected many people at the time of their peak, but after the diseases regressed, limited reports of laboratory accidents or animal-to-human transmission were published.

Until today, no treatments have been reported for COVID-19 (7). Although some case-reports or observational studies have reported a few antiviral drugs being effective in improving the outcome of COVID-19 patients (8), no definitive cure has been discovered so far. Recently, a Chinese research team proposed a treatment protocol for management of COVID-19 patients, which included moxifloxacin, levofloxacin (consider tolerance) and arbidol administration (8). However, the underlying evidence for the mentioned treatment protocol is unclear, as there are no clinical trials performed on this matter. Several antiviral therapies have been considered to be potentially effective in treating COVID-19, including oseltamivir, ganciclovir, arbidol and lopinavir / ritonavir. Many clinical trials are currently underway to evaluate the efficacy of different medications on the outcome of COVID-19 patients (9), but their results have not been published yet. On the other hand, the quality of these studies is rather unclear.

Treatments used for SARS-CoV or MERS-CoV may be useful in the treatment of COVID-19, due to the fact that all of these viruses are from the same family, and they all cause respiratory diseases. Nevertheless, a consensus is yet to be reached on this matter. The primary objective of the current systematic review is to evaluate the evidence underlying the efficacy and safety of antiviral therapies in treatment of COVID-19 in the current pandemic. Another goal for the present study is to investigate the clinical trials performed in recent years on the effects of antiviral therapies on SARS-CoV or MERS-CoV to propose potential existing treatments for COVID-19.

## Methods

**Study design**

The present systematic review was performed in two sections. In the first section, current clinical evidence about the efficacy of antiviral treatments in management of COVID-19, namely the COVID-19 antiviral therapy section, is presented. The second part provides a review of clinical trials conducted on SARS-CoV and MERS-CoV to find proposed antiviral therapies, namely the SARS-MERS antiviral therapy section.

**Selection criteria**

In the COVID-19 antiviral therapy section, all types of performed clinical studies, aiming to evaluate the efficacy and safety of antiviral drugs were included. Exclusion criteria comprised in vitro studies, animal studies, guidelines, and review studies.

In the SARS-MERS antiviral therapy section, inclusion criteria consisted of clinical trials on the efficacy and safety of antiviral drugs administered for management of ARS-CoV and MERS-CoV. The exclusion criteria in this section contained observational studies, guides, animal studies, and review articles. Since SARS and MERS outbreaks happened in recent years, and there was the opportunity for conduction of clinical trials, only clinical trials were included in this section, as they provide the highest level of evidence. In this section, antiviral therapies for COVID-19 are to be suggested, so the provided underlying evidence should be of highest validity.

**Search strategy**

An extensive search was performed on Medline (via PubMed), Embase, Scopus, CENTRAL, and Web of Science databases. The keywords were selected using expert opinions, Mesh, Emtree and related article titles. 

The keywords in the COVID-19 antiviral therapy section were the only keywords associated with COVID-19. Since a small number of articles were published about the treatment, and the use of antiviral treatments were prevalently not mentioned in the abstracts of the articles, keywords related to “antiviral therapy” were not included in the search. Also, the search date was chosen to be from early 2019 until March 15, 2020, since the first report of COVID-19 was published in late 2019.

The keywords in the SARS-MERS antiviral therapy section included keywords related to SARS-CoV and MERS-CoV in combination with standard filters for clinical trials. Similar to the previous section, keywords related to antiviral therapy were not included in the search, due to the limited number of studies. The time range for search was set to be from the inception of the database until March 15, 2020. Search strategy in Medline is presented in Table 1. In addition to the systematic search, an extensive search was also performed on google and google scholar search engines and in the article bibliographies.

**Data collection**

Two independent researchers reviewed the titles and abstracts of the records obtained from the databases and selected related articles. Next, full texts of these articles were collected and reviewed carefully. Finally, related articles suitable based on inclusion and exclusion criteria were included in the present systematic review. Then, the two researchers summarized the articles and collected data including the name of the first author, publication year, country in which the study was conducted, type of the study, sample size, age and sex distribution of the patients, the drug used, dose and duration of administration, route of administration and treatment outcome. Any disagreements were resolved by a discussion with a third researcher.

## Results

**Studies on COVID-19**

**Characteristics**

**Figure 1:**
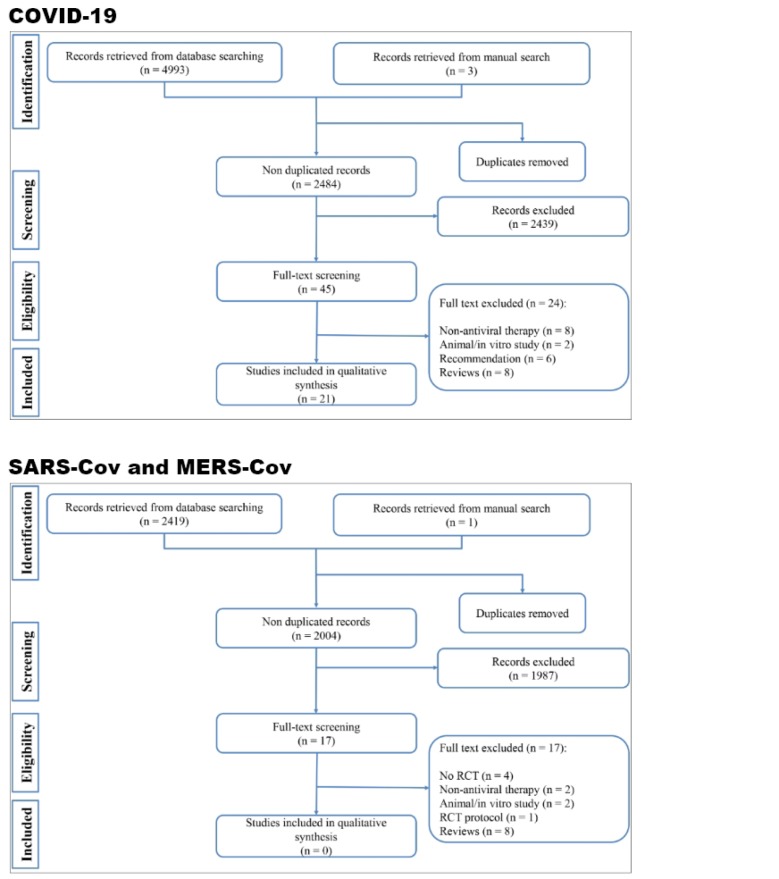
Flow diagram of screening process of present systematic review. RCT: Randomized clinical trial.

The initial search yielded 4997 articles. After eliminating duplicates, 2485 records were reviewed, and 22 studies were included in the current systematic review (Figure 1) (10-31). 20 articles were conducted in China, one article was conducted in South Korea and one study was performed in Singapore. Only one clinical trial was found, which evaluated the efficacy of Lopinavir-Ritonavir in treating patients with severe COVID-19 (10). Among the included articles, 16 were case-series (11-13, 15, 16, 19-23, 25-29, 31) and 5 were case-reports (14, 17, 18, 24, 30). The articles studied 2856 COVID-19 patients, 1883 (65.9%) of whom were treated with antiviral agents. The most commonly used antivirals were lopinavir / ritonavir, oseltamivir, ribavirin and arbidol, respectively. Route and duration of administration were not reported in most of the studies. The most common route of administration among the studies was oral administration, and the duration of administration varied from two to 14 days. All of the studies used other therapies, such as antibiotics, immunoglobin, interferon, glucocorticoids, methylprednisolone, and antiparasitic and antifungal drugs in addition to the antiviral therapy to manage COVID-19 patients (Table 2).

**Antiviral therapy for management of COVID-19**

The only clinical trial found was performed on 199 patients with severe COVID-19, using Lopinavir-Ritonavir regimen. In this randomized open-labeled clinical trial, patients were divided into two groups: lopinavir-ritonavir (99 patients) group and standard treatment group. In addition to lopinavir-ritonavir (400 mg / 100 mg, twice daily; 14 days), standard treatments such as antibiotics, invasive or non-invasive ventilation and extracorporeal membrane oxygenation (ECMO) and vasopressor were also used. The findings of this study showed that patients receiving lopinavir-ritonavir had a similar recovery process to those receiving standard treatment. 28-day mortality and viral RNA load were not significantly different between the two groups (10). The researchers concluded that lopinavir-ritonavir administration in patients with severe COVID-19 was not more effective than the standard treatment.

**Table 1:** Search queries in Medline database

**Section**	**Search query**
COVID-19 antiviral therapy	Coronavirus[tiab] OR COVID19[tiab] OR 2019-nCoV[tiab] OR COVID19 virus[tiab] OR COVID-19 virus[tiab] OR 2019-nCoV disease[tiab] OR 2019 novel coronavirus disease[tiab] OR 2019-nCoV infection[tiab] OR 2019-nCoV[tiab] OR Coronavirus disease 2019 virus[tiab] OR SARS-CoV-2[tiab] OR SARS2[tiab] OR 2019 novel coronavirus[tiab] OR 2019 novel coronavirus infection[tiab] OR coronavirus disease 2019[tiab] OR coronavirus disease-19[tiab] OR new coronavirus[tiab] OR Wuhan coronavirus[tiab] OR Wuhan seafood market pneumonia virus[tiab] OR "severe acute respiratory syndrome coronavirus 2"[Supplementary Concept] OR "COVID-19"[Supplementary Concept]
SARS-MERS antiviral therapy	1. “Coronavirus”[mh] OR “Betacoronavirus”[mh] OR “Middle East Respiratory Syndrome Coronavirus”[mh] OR “SARS Virus”[mh] OR Coronavirus[tiab] OR Coronaviruses[tiab] OR Deltacoronavirus[tiab] OR Deltacoronaviruses[tiab] OR Munia coronavirus HKU13[tiab] OR Coronavirus HKU15[tiab] OR Coronavirus, Rabbit[tiab] OR Rabbit Coronavirus[tiab] OR Coronaviruses, Rabbit[tiab] OR Rabbit Coronaviruses[tiab] OR Bulbul coronavirus HKU11[tiab] OR Thrush coronavirus HKU12[tiab] OR Betacoronavirus[tiab] OR Betacoronaviruses[tiab] OR Tylonycteris bat coronavirus HKU4[tiab] OR Pipistrellus bat coronavirus HKU5[tiab] OR Human coronavirus HKU1[tiab] OR HCoV-HKU1[tiab] OR Rousettus bat coronavirus HKU9[tiab] OR Middle East Respiratory Syndrome Coronavirus[tiab] OR MERS-CoV[tiab] OR MERS Virus[tiab] OR MERS Viruses[tiab] OR Virus, MERS[tiab] OR Viruses, MERS[tiab] OR Middle East respiratory syndrome-related coronavirus[tiab] OR Middle East respiratory syndrome related coronavirus[tiab] OR SARS Virus[tiab] OR Severe Acute Respiratory Syndrome Virus[tiab] OR SARS-Related Coronavirus[tiab] OR Coronavirus, SARS-Related[tiab] OR SARS Related Coronavirus[tiab] OR SARS-CoV[tiab] OR Urbani SARS-Associated Coronavirus[tiab] OR Coronavirus, Urbani SARS-Associated[tiab] OR SARS-Associated Coronavirus, Urbani[tiab] OR Urbani SARS Associated Coronavirus[tiab] OR SARS Coronavirus[tiab] OR Coronavirus, SARS[tiab] OR Severe acute respiratory syndrome-related coronavirus[tiab] OR Severe acute respiratory syndrome related coronavirus[tiab] OR SARS-Associated Coronavirus[tiab] OR Coronavirus, SARS-Associated[tiab] OR SARS Associated Coronavirus[tiab] 2. (randomized controlled trial [pt] OR controlled clinical trial [pt] OR randomized [tiab] OR placebo [tiab] OR drug therapy [sh] OR randomly [tiab] OR trial [tiab] OR groups [tiab]) NOT (animals [mh] NOT humans [mh]) 3. #1 and #2

**Table 2:** Clinical studies that reported anti-viral therapy in management of COVID-19

First author; Year; Country	Sample size	Age (year)*	Male	Status of patients	Antiviral agent	Antiviral treated patients (n)	Dosage	Route of administration	Duration of treatment	Combination	Main findings
**Randomized clinical trial**											
Cao et al; 2020; China (10)	199	58 (50 to 68)	120	Severe COVID-19 patients	Lopinavir/Ritonavir	99	400 mg/100 mg twice daily	Oral	14	Yes	Lopinavir–ritonavir administration is not superior to standard care in management of adult patients with severe COVID-19. Clinical improvement and mortality rate are similar in lopinavir–ritonavir treated and standard care groups.
Case-series											
Chen N; 2020; China (11)	99	21 to 82	67	COVID-19 patients	Oseltamivir	75	75 mg twice a day	Oral	3 to 14	Yes	Recovery rate: 31%; Mortality rate: 11%
Chen Q; 2020; China (12)	9	14 to 56	5	Symptomatic COVID-19	Lopinavir/ Ritonavir	9	800 mg/200 mg daily	Oral	4 to 11	Yes	No mortality. Time from onset of treatment to negative result of Cov-test was 4-11 days. Length of hospital stay was 9 to 20 days.
Guan W; 2020; China (13)	1099	47.0 (IQR: 35.0–58.0)	637	Non-severe and severe COVID-19 patients	Oseltamivir	393	NR	NR	NR	Yes	Administration of oseltamivir did not decrease ICU admission and need for ventilator or death
Hu Z; 2020; China (15)	24	5 to 95	8	Asymptomatic COVID-19 infection	Not specified	21	NR	NR	NR	Yes	No mortality, no ICU admission, no severe complication
Huang C; 2020; China (16)	41	49 (IQR 41·0–58·0)	30	Symptomatic COVID-19	Oseltamivir	38	NR	NR	NR	Yes	6 patients died 28 patients were discharged
Liu K; 2020; China (19)	137	20 to 83	61	Severe COVID-19	Not specified	105	NR	NR	NR	Yes	16 patients died during the study.
Liu L; 2020; China (20)	51	16 to 68	32	Discharged COVID-19 patients	Lopinavir/ Ritonavir Oseltamivir Arbidol	51 7 2	NR	Oral	NR	Yes	Duration of hospital stay was 9-13 days. 1 patient died.
Qin X; 2020; China (21)	89	23 to 86	45	All COVID-19 patients admitted to a center	Lopinavir/Ritonavir Other anti-viral	84 5	NR	NR	NR	Yes	16 patients were discharged and 1 patient died.
Shang J; 2020; China (22)	416	49 (IQR: 36-61)	194	Survived and dead COVID-19 patients	Not specified	380	NR	NR	NR	Yes	Anti-viral administration did not affect mortality rate (5.6% in non-treated vs. 12.9 treated; p=0.288)
Wang D; 2020; China (23)	138	22 to 92	75	ICU and Non-ICU admitted COVID-19 patients	Oseltamivir	124	NR	NR	NR	Yes	6 patients died and 36 patients were admitted to ICU.
Wu J; 2020; China (25)	80	46.10 ± 15.42	39	All severity ranges of COVID-19	Ribavirin	80	NR	NR	2-12 days	Yes	21 patients were discharged and 59 patients remained in hospital.
Xu X; 2020; China (26)	62	41 (IQR: 32-52)	35	Symptomatic COVID-19	Lopinavir/ritonavir Arbidol Lopinavir/ritonavir + Arbidol	25 1 21	Lopinavir 400 mg twice daily ritonavir 100 mg twice daily Arbidol 200 mg three time daily	NR	NR	Yes	One patient was discharged. Other patients remained in hospital
Yang W; 2020; China (27)	149	45.11 ± 13.35	81	All COVID-19 patients admitted to a center	Not specified	140	NR	NR	NR	Yes	No mortality. 73 patients were discharged and 76 remained in hospital.
Young BE; 2020; Singapore (28)	18	31 to 73	9	Symptomatic COVID-19	Lopinavir/ritonavir	5	NR	NR	NR	Yes	Two patients recovered and the condition of 2 other patients deteriorated. Only one patient completed the 12-day planned protocol. Four patients experienced side effects of antiviral therapy
Zhang G; 2020; China (29)	221	20 to 96	108	Non-severe and severe confirmed COVID-19 patients	Not specified	196	NR	NR	NR	Yes	12 patients died. Chest CT improved after administration of ECMO and IMV
Zhou Z; 2020; China (31)	10	29 to 68	8	Confirmed COVID-19 patients	Lopinavir/ritonavir Arbidol	8 3	NR	Oral	NR	Yes	1 patient died, 5 patients remained hospitalized and 4 patients were discharged
Case reports											
Han X; 2020; China (14)	1	23	1	Diabetic patient with COVID-19	Oseltamivir/ Gancivlovire	1	NR	NR	15	Yes	Patient was discharged from hospital after 15 days
Li W; 2020; China (17)	5	10 months to 6 years	4	Children with COVID-19	Not specified	2	NR	NR	NR	Yes	The antiviral therapy did not change the outcome or length of stay
Lim J; 2020; South Korea (18)	1	54	1	Symptomatic COVID-19 patient	Lopinavir/ritonavir	1	75 mg twice a day/50 mg twice daily	Oral	9	Yes	Good recovery. It is not clear that the decreased load of virus is due to the nature of healing process or a result of anti-viral therapy
Wang Z; 2020; China (24)	4	19 to 63	3	COVID-19 patients	Lopinavir/ritonavir Arbidol SFJDC	4	400 mg/100 mg twice daily 0.2 g, three time daily 2.08 g, three time daily	Oral	6-16 days	Antibiotic	2 patients recovered and 2 patients remained in hospital
Zhang Z; 2020; China (30)	2	38	1	Symptomatic COVID-19 patients	oseltamivir and Arbidol	2	NR	NR	NR	Yes	Both patients recovered and were discharged

*,Age was reported as range, mean±SD or median (interquartile rang [IQR]). ECMO: extracorporeal membrane oxygenation; IMV: invasive mechanical ventilation; NR: Not reported; ICU: intensive care unit; CT: computed tomography

As previously mentioned, all of the other studies were case-reports and case-series. Moreover, most of the studies did not provide an analysis regarding the efficacy of antiviral therapy in the treatment course of COVID-19 patients. For instance, Chen et al. reported no deaths among nine patients treated with Lopinavir/Ritonavir. However, the sample size of the mentioned article was quite small (12). According to the existing statistics, the mortality rate of COVID-19 among patients aged 10 to 60 years, varies between 0.2% and 1.3% (3). Hence, for every 100 patients in this age group, only one patient dies. Therefore, the sample size was probably not large enough to observe at least one death. In another study, Hu et al. studied 24 asymptomatic patients and reported no mortality or ICU admission, and no serious complication in 21 patients treated with antiviral agents. Nonetheless, in addition to the small sample size, since the patients were asymptomatic, the severity of COVID-19 was rather mild in them. Therefore, the recovery of the patients might have been due to their mild disease, rather than the efficacy of the antiviral drugs used (15). 

Guan et al. studied 1099 patients, 393 of which were treated with oseltamivir and showed that ICU admission, need for mechanical ventilation, or death rate was 9.2% among the oseltamivir-treated group, whilst the rate was 4.4% in the group that was not treated with oseltamivir. In other words, oseltamivir administration was ineffective in decreasing ICU admission rate, the need for ventilator and death rate among the patients (13). Shang et al. performed a study on 416 patients, indicating that antiviral drugs have no effects on the mortality rate of COVID-19 patients. In their study, the mortality rate was 12.9% among the group treated with antiviral drugs, whereas the group which did not receive the antiviral treatment had a mortality rate of 5.6% (22). Furthermore, in Singapore, Young et al. studied 18 patients, and administered lopinavir/ritonavir for only five patients, two of whom recovered and two deteriorated. Only one patient tolerated the antiviral regimen and completed the 12-day treatment course. After the start of antiviral treatments, liver test results became abnormal in three of the patients (28).

Also, Li et al. examined five children with COVID-19 (two patients treated with antiviral drugs and three patients did not receive antiviral therapy), indicating that antiviral agents did not change the outcome or the duration of hospital stay (17). Lim et al. presented a 54-year-old non-smoker woman with no medical history, in whom the viral load started to decline and symptoms started to alleviate gradually with the start of lopinavir/ritonavir treatment, and eventually, the patient was discharged. The researchers reported that it is not clear that the reduced burden of COVID-19 is due to the healing process or to the administration of anti-viral therapy (18). Furthermore, Han et al. and Zhang et al. reported complete recovery in one patient who had diabetes and was treated with oseltamivir/ganciclovir (14) and two patients treated with oseltamivir and arbidol (30), respectively.

There are also studies available that, despite having an acceptable sample size, did not report the final outcome of a large portion of their patients. For instance, Wu et al. did not report any deaths among 80 patients treated with ribavirin, while 59 patients were still not discharged and their outcome was still unclear at the time of the submission of the article (25). Xu et al. (26), Yang et al. (27) and Wang et al. (24) also observed no deaths in their studies. However, the majority of the patients they reviewed were still under treatment, with an unclear final outcome, at the time of the publication of their articles.

**Studies on** **SARS-CoV and MERS-CoV**

An extensive search was conducted in databases, in order to find the existing evidence regarding antiviral therapies for treatment of diseases caused by SARS-CoV and MERS-CoV, which are in the same family as SARS-CoV-2. In this section, only clinical trials were supposed to be found. 2004 non-duplicate articles were reviewed, and no clinical trials were performed on the antiviral treatments in managing SARS-CoV and MERS-CoV patients (Figure 1). Only one clinical trial protocol commissioned by a research team in Saudi Arabia existed. This study, named as the MIRACLE trial, was designed to evaluate the efficacy of lopinavir / ritonavir and interferon-β1b combination therapy in the treatment of MERS-CoV patients. The study is currently ongoing, and their results have not been published yet (32). Therefore, no clinical trials exist on the basis of which an antiviral drug can be suggested for COVID-19, not even for SARS-CoV and MERS-CoV.

## Discussion

The findings of the present study indicated that only one clinical trial was completed on the efficacy and safety of antiviral agents in management of COVID-19 patients, which showed ineffectiveness of lopinavir-ritonavir in improving patients’ outcomes. Moreover, no clinical trials exist on treatments for SARS-CoV and MERS-CoV. Furthermore, in 2019 and 2020, 21 case-series and case-reports provided reports about antiviral treatments in management of COVID-19 patients, none of which can be taken into account when assessing the clinical usefulness of antiviral treatments against COVID-19. In general, no study has examined the efficacy of antiviral therapies alone in treatment of COVID-19, and all of the existing articles have used other treatments such as antibiotics, immunoglobulin, interferon, glucocorticoids, antifungal and antiparasitic drugs in their studies. Hence, the reports presented cannot be attributed solely to antiviral drugs. Therefore, the existing literature regarding the efficacy of antiviral therapy in management of COVID-19 patients has serious limitations including:

1. Only one clinical trial has been conducted on the matter, depicting that lopinavir-ritonavir is not superior to the standard treatments in management of COVID-19 patients.

2. In all of the existing case-series and case-reports, antiviral agents were used in combination with other medications, and therefore the observed outcomes cannot be solely attributed to antiviral therapy.

3. Since there was no placebo group in the case-series and case-reports, one cannot determine that the outcomes are due to antiviral therapy or the nature of healing process or both.

4. Follow-up was incomplete in some studies, and a great proportion of the patients have unclear outcomes.

5. The sample size was small in most of the studies, and some were even case reports.

Recently, Zhang et al. posted a guideline in the Lancet Respiratory Medicine journal, recommending the administration of Arbidol for management of COVID-19 patients. However, this guideline has limitations. In general, the underlying evidence for this guideline are unclear. Secondly, based on the present systematic review, arbidol was used as the second line of therapy in all of the included studies. Hence, the drug appears to be inappropriate for the first line of therapy (8). Nevertheless, lopinavir-ritonavir being ineffective in management of COVID-19 patients highlights the need to search for other antiviral therapies.

Many existing guidelines and instructions propose antiviral drugs for management of COVID-19 patients, based on the studies performed on SARS-CoV and MERS-CoV. Therefore, in the current study we aimed to summarize the evidence obtained from clinical trials to determine the level of evidence underlying the mentioned proposal. Surprisingly, no completed clinical trial on SARS-CoV and MERS-CoV was found, and all of the proposals were based on simulation studies, in vitro studies, pre-clinical studies or at best, observational studies and case-series (33-38). After an epidemic has subsided, researchers are reluctant to perform further investigations on the matter, which is a major pitfall in research and can have several reasons. The first and most important reason is the decline in the number of patients, which severely limits the possibility of performing a clinical trial. Secondly, the previous two epidemics, namely SARS-CoV and MERS-CoV, never became a pandemic, and the majority of the cases were in a limited geographic region. Therefore, after the suppression of the epidemics and decline in the number of patients, the two diseases were eliminated from the list of priorities of international organizations. If those international organizations, such as the World Health Organization, had further insisted on the importance of the subject and provided grants for independent researches, maybe a few effective antiviral agents for SARS-CoV and MERS-CoV would have existed, which could be helpful in management of COVID-19, decreasing its burden on societies. A lesson should be learned from the COVID-19 pandemic and negligence of health policy makers in setting up clinical trials during SARS-CoV and MERS-CoV epidemics, so that in the current pandemic and future epidemics, researches and well-designed clinical trials are planned-out while the disease is at its peak, the findings of which can be used when necessary.

## Conclusion

Findings of the current systematic review indicated that it is not clear whether the currently used antiviral agents are beneficial in improving the outcome of COVID-19 patients or not. One clinical trial and some case-series suggest that these drugs may not have an impact on the final outcome of COVID-19 patients. Furthermore, lack of published clinical trials on SARS-CoV and MERS-CoV, which were epidemics in recent years, impedes suggesting a potential antiviral treatment for COVID-19. The current situation is a serious red flag for international organizations such as the World Health Organization. In the time of the current pandemic and future epidemics, organizations such as WHO should pursue more proactive actions and plan well-designed clinical trials to be able to use the results in managing future pandemics.
